# Diseases of the Maxillary Sinus

**Published:** 1856-10

**Authors:** J. A. Giraldes, Christoper Johnston

**Affiliations:** Professor agrégé à la Faculté de Medecine, &c. &c., Paris.; Baltimore.


AMERICAN JOURNAL

OF

DENTAL SCIENCE.

Vol. VI. NEW SERIES—OCTOBER, 1856. No. 4.

### ORIGINAL COMMUNICATIONS.

### ARTICLE I.

*Diseases of the Maxillary Sinus.* By DR. J. A. GIRALDES, Professor agrégé à la Faculté de Medecine, &c. &c., Paris. Translated by DR. CHRISTOPER JOHNSTON, Baltimore.

(Continued from page 417.)

### SECTION IV.—*Modification in Nutrition with formation of new products.*

*Solid Tumors.*—The question that we are about to treat, one of the most important of our subject, one of the most curious of pathology in general, is also one of those to which the least attention has been directed; wherefore it is not surprising that we should find much confusion in regard to this matter in works which comprehend it in their scope. In fact, the diseases under consideration have always been confounded together under the generic name of tumors, and consequently *clinicians* have ranged the most dissimilar things in the same category: under the capitulation of fungus, cancerous tumors, they have grouped entirely different products and diseases, unlike in their anatomical constitution, and, what is worse, in the various consequences to which they give rise. This point of our subject must be studied anew, and the study cannot be prosecuted simply with the united observations in the annals of the art, but we must add to them a clinical analysis carefully aided by microscopic observation and the other means which science now employs. In a clinical point of view this study is of the first importance, for it only can enlighten the practitioner as to the gravity of the affection and the consequences which may ensue; it alone may justify great operations, which are the honor of surgery when they are conceived and done under suitable conditions: and, on the other hand, may it authorize the complete rejection, in certain cases, of the intervention of operative medicine.

The tumors met with in the antrum of Highmore, and which most frequently invade the tissues of the face, may be classified, as well as all morbid productions, under two distinct heads: the first comprising tumors having some analogy with the normal tissues, and the second including tissues of abnormal constitution not otherwise discovered in the organism. In the first category are placed *fibrous*, *cartilaginous*, *osseous tumors*, as well as *enchondromata* and *erectile* and *epithelial tumors*. In the second are grouped *fibro-plastic tumors* and those of *cancerous nature.**

§ 1. *Tumors formed of tissues having their analogue in the economy.*—The tumors forming this division are: 1st, Fibrous, fibro-cartilaginous, fibro-cystic, enchondromatous, osseous, erectile, epithelial and fatty.

Before going farther in this study it must be stated that these productions are frequently developed in the sinus itself, and that, at times, they originate not only in the sinus, but also in

* The translator begs leave to say, that this division of the subject, for which Lebert was originally responsible, is inexact in so far as concerns the *epithelial*, the *cancerous* and the *fibro-plastic* elements. The last named are admitted by C. Robin, (Tableaux d'Anatomie, &c.) and Lebert to be normal constituents and pathologically *homœomorphous*—and Müller and Velpeau, having long denied the existence of a *specific microscopic element of cancer*, and the truth of observation having forced M. Robin to a confession of their faith, the microscope appears to be no longer diagnostical of cancer and of epithelioma; and if any differences exist they must be established upon other grounds than the *dicta* of that instrument.

the surrounding parts, from which they sometimes take new elements, and add them to their own. Thus, in certain cases, the periosteum of the bones of the face, and other tissues, become unusually vascular, the periosteum is the seat of ossification and contributes to solder to neighboring parts the tumor originally developed in the maxillary sinus, and to form a mass which it is oftentimes difficult to trace to its initial point; thus Peele relates an example of an osseous tumor occupying the antrum of Highmore developing itself towards the anterior part of the face, extending itself into the cranium, but of which it is impossible to understand the origin; it is of the same class as those described by Howship;* as those of which Bordenave has given a description;† of these enormous exostoses found in the Dupuytren museum, and of another identical production of the same kind in the Hunterian museum, labelled 3236.

If tumors developed in the sinus can be confounded with the other morbid productions of the bone of the face so far as to render the precise distinction of their origin doubtful, it would be those formed without the maxillary sinus surrounding or depressing its cavity. Stanley‡ cites the case and gives the drawing of a tumor developed in the gums, which established itself in the cavity of the sinus by forcing before it and surrounding the osseous laminæ. Lyne§ relates an instance of a very curiously developed tumor penetrating into the sinus in the same manner. Thus this distinction of tumors developed in the sinus or penetrating into its cavity is not a division purely theoretical, it will add very great interest to their clinical study.

*Fibrous Tumors.*—Tumors of a fibrous nature are to be found within the maxillary sinus. Lisars,|| Warren,¶ Burgrave,** Lupuytren,†† Knor‡‡ and Lubourg§§ have given cases. The anatomical constitution of these tumors has not been studied

* Ostiosarcour ejusque Specici ensequis descriptio. Wirceb, 1819.

† Practical Obs. on Sur. and Mort. Anatomy.

‡ On Diseases of the Bones; illustrat. pl. 16.

§ London and Ed. Monthly Journal, 1843, June, page 1.

|| Practical Surg. ¶ Surgical Obs. on Tumors. Boston, 1848, p. 480

** Gaz. des Hos., 1849, p. 61. †† Lecons Clin., vol. 3.

‡‡ Soc. cit. §§ Bull, de la Soc. Anat., 1828.

with care; as far as we have observed, authors who have had occasion to examine these tumors have confined themselves to a mere account of their nature, saying only that they were formed of a white and reticulated fibrous tissue, &c. Tumors of this kind seem, according to O'Shaughnessy,* quoted by Stanley, to be common in the Indies, and in this country they grow to a considerable size. This author gives an example of a tumor of the maxillary sinus weighing four ounces. He adds, they are more frequently observed among the young Indians.

Fibrous tumors of the maxillary sinus are met with in persons of all ages; the causes which determine their formation are but little known. In some cases, observation by M. Bergrave, the origin of the disease is attributed to a contusion; but this etiology is far from being demonstrated, and knowing that blood poured out at one time does not undergo the same change that it does at another, the coincidence of a contusion with the production of one of these tumors is of but little importance in explaining its development. Fibrous tumors of the maxillary sinus attain to a very great size; during their development they distend the walls of this cavity, and, like the productions of another species, they attack and surround the adjoining parts, and are confounded with them. Sometimes, however, they penetrate the sinus at a fixed point. Thus, Warren gives a case of an individual, aged fifty-one years, in whom a tumor of this kind had extended to the lower eyelid, protruding exteriorly and forming a bulbous mass, believed to be a fibrous tumor of the eye lid. The tumor having been removed, it was seen to have grown from the antrum and protruded through an opening of this cavity. Trennery has seen a case in which the fibrous mass invaded the entire face.† Tumors of this kind sometimes present osseous spicula; Leiton gives an example.

*Fibro-Cartilaginous Tumors.*—As the tumors previously described, those of a fibro-cartilaginous nature have been supposed, from an inspection with the naked eye, to present this structure,

* Vide Lancet, 1850, p. 574. † Medic. Chir., trans., t. xx.

and even at the present day, no other distinction has been demonstrated. Lisars, Liston and Gensoul, give examples of such tumors. Morbid growths of this kind are ordinarily of a rounded form; the name is at once suggested by their texture. Some, for example, as those of which Liston speaks, are formed of white fibrous matter mixed with gelatinous substance, coagulable by heat and enclosed in cells. In one case, the treatment of which I had charge in the hospital of la Pitié, in 1847, the tumor presented a white rounded mass, having the aspect of cartilage and formed of cells similar to those in the honey-comb, filled with gelatinous matter.

Fibro-cartilaginous tumors sometimes attain to a very large size. In the case described by Gensoul, the tumor was seven inches in diameter.

*Fibro-cystic Tumors.*—In some cases of fibrous tumors of this cavity, cysts are found in their substance. Cruveilhier,* examined, after death, the case of a woman aged sixty-eight years, in the maxillary sinus of whom there existed a voluminous fibrous tumor filled with hollow cysts.

*Enchondroms.*—I have never met with a case of enchondromatous tumors of the maxillary sinus, but think it likely that some morbid growths described under the name of fibro-cartilaginous tumors might belong to this class. This supposition is rendered probable as enchondroms are not of rare occurrence and have been frequently confounded with cartilaginous exostoses. Besides, tumors of this kind have only been studied since the publication of the work of J. Müller, and particularly since the patient labors of M. Luckett. This able and learned anatomist has examined the structure of enchondroms with much care, and has added his researches to the catalogue of microscopic preparations of the Hunterian Museum.

*Progress and Diagnosis.*—The tumors now under consideration are usually of rapid growth and have a tendency to invade the surrounding parts. Before bursting the walls of the cavity in which they are situated, they extend in every direction; they

* Essays upon Ant. and Path., vol. 1, p. 393.

also tend during their evolution to shrink and destroy the cavity formed in the maxillary bone. It is thus that the floor of the orbit is elevated and the eye forced out of its cavity; that the nasal cavity is filled and obliterated by the development of the morbid mass; that the vault of the palate is depressed, forming a round tumor; that the depressions of the canine fossæ enlarge and disappear, and that the dental arch becomes embedded in the side of the tumor. But these modifications are not always observed in the periphery. Whilst this is going on the bone becomes thin, and when pressed feels like rumpled parchment.

It is easily understood that a growth of this nature, obliterating and destroying the cavity connected with such important nerves, could not exist without being accompanied with sympathetic phenomena, such as sharp and intense pain of the face and side of the head. It is also observed that vision is perverted or completely destroyed; the sense of hearing is sometimes impaired by the compression of the eustachian tube or by some other cause. These tumors have a tendency to affect the surrounding tissues, causing increased vascularity, thickening and inflammation of the mucous membrane—having sometimes upon their surface bloody fungi, which are often attended with serious consequences. The skin of the face itself does not always escape these changes; it becomes vascular, the veins dilate, assuming a varicose condition.

*Prognosis.*—The prognose of these tumors is directly connected with the precise knowledge of their nature and of their histological composition. Those designated by the name of fibrous, fibro-cartilaginous, &c., when they are really formed by elements which seem to indicate their character should not be considered as serious. In fact they are removed without any serious consequences to the patient, and he recovers without the slightest constitutional impairment. What is most satisfactory and important to know is, that these tumors are never reproduced.

*Osseous Tumors.*—Osseous tumors of the maxillary sinus have been observed by David, Hundertmack, Otto, Georgi and M. Lenoir, &c. Tumors of this description belong to two distinct classes. Some are found in the sinus itself: M. Michou has met with a very remarkable example which he presented to the Chirurgical Society. Others occupy at the same time the cavity and surrounding osseous tissues, as has been shown by David, Buttner, M. A. Pettit, Warren, M. M. Huguier and Hancock.

Tumors of this kind are almost always accompanied during the progress of their development with deposits of osseous matter in the periosteum and in the maxillary bone. There is developed in the facial bones a true osteosis—a bony envelop enclosing a new production—an *osteide.* It constitutes an almost stony mass, recalling the form of the osteide in the ear of cetaceans. These masses are of an ivory whiteness; they have a fluted form, sometimes with a series of globules united between them, a very compact and hard texture and are very heavy. Their microscopic texture is similar to that of bony tissue. A piece of this kind which I have before me, and for which I am indebted to Dr. Follin, found in the sinus of a horse, shows the periosteum to be covered with osseous productions of new formation resembling needle-like crystals, amongst which are dispersed particles analogous to manna in flakes.

The second kind of osseous tumors occupy both the sinus and the maxillary bone. Ruttner gives an example of a case of this description. South notices a case, the preparation of which is in St. Thomas' Hospital, and Hancock describes a bony tumor weighing seven ounces, occupying the upper jaw and antrum. This second description of tumor belongs to the class of spongy exostosis. The structure is alveolar and easily penetrated by a cutting instrument, the meshes are filled with a reddish substance slightly analogous to spongy bone. M. A. Pettit, Gensoul, Warren, M. M. Huguier and Lenoir, notice tumors belonging to this class. They invade the face as well as the maxillary sinus; they sometimes attain to a considerable size, as the observations of Bordenave and Hundertmack show. They constantly augment and develop themselves either from the side of the orbit, nasal fossæ or alveolar arch, which, by the compression they exert upon the neighboring parts, are often productive of serious consequences, as is shown by the case quoted by David.

The causes concerned in the production of these tumors are not well understood. It is probable they sometimes owe their origin to a syphilitic vice. Bayer mentions a case of a large exostosis occupying the jaw and extending to the side of the orbit, having all the characteristics of the morbid growths now under consideration, and which disappeared under the influence of anti-syphilitic remedies.

*Prognosis.*—From the tendency to enlarge and develop themselves in the cavities and to interrupt the functions of the mouth, nose and eyes, their prognosis is unfavorable.

*Erectile Tumors.*—Erectile tumors of the maxillary sinus are very rare. We do not remember to have met with a case, but believe the one described by Gensoul as a varicose tumor may be classed under this head. There is another description of tumor of this cavity, which Pattison thinks is of the nature of those now under consideration. It is described in the American edition of Burns' Surgical Anatomy. In this case the carotid artery was tied and the patient recovered. As these tumors are of rare occurrence, and as but few autopsies have been made, we must be cautious in admitting the existence of erectile tumors in this cavity. The remarks of Delpch would seem to render such caution necessary. This surgeon met with a tumor of the maxillary sinus in a man fifty years of age, presenting all the characteristics of an erectile tumor. Pulsation, susurrus, shrinking of the tumor by compression of the carotids; in fine, a series of symptoms appearing sufficient to determine its erectile character. The patient died, and the autopsy showed the tumor to be of any other nature than erectile.

*Fatty Tumors.*—This description of tumors is not described by authors as occurring in the maxillary sinus. Stanley speaks of them, but does not give a description; he only states in a note that they are formed of globules of stearine. Tumors of this kind must be very rare, as we have no other mention of them.

M. Viard presented to the Anatomical Society a case of fatty tumor of the maxillary sinus, of which the following is the description: "The superior maxillary of the right side exhibits an enlargement of the size of a turkey's egg, and the bone is diseased throughout the whole extent, yielding easily to pressure. A fatty mass has almost entirely taken the place of the bone. It completely fills the cavity of the maxillary sinus, but the disease does not seem to have originated in this cavity, for in the substance of the tumor there are bony laminæ intersected and separated one from another by adipose tissue." (15 *Mai.* 1850. *Bul. Soc. Nat.*)

SECTION II.—*Tumors formed of a Normal Tissue—Heterologous Tumors.*—This class of tumors includes those most frequently met with in the maxillary sinus; they also attack the greater portion of the maxillary bone, and are usually preceded by a pathological condition of the sinus. We find in the study of these tumors the same confusion that attends the study of those belonging to the first category; thus, under the name of encephaloid and cancerous, we confound tumors of various nature and of different degrees of malignancy. Fibro-plastic, epithelial, and encephaloid tumors are all confounded under the vague denomination of cancer of the jaw.

In this category of tumors we should establish two distinct classes. In the one fibro-plastic, and the other encephaloidal tumors. I will not attempt to describe the microscopical elements peculiar to each of these classes. I can only say that clinical observation seems to justify the division I have established; the first or fibro-plastic tumors are seldom liable to be reproduced in any form, whilst the second, encephaloidal, have a fatal tendency to return after their removal. It only remains to examine the degree of frequency of each, as well as the period of life in which they are most frequently developed, but this branch of pathological inquiry has not been very thoroughly investigated.

We establish two classes of heterologous tumors: first, fibro-plastic, second, encephaloidal, but will not give a separate description. Heterologous tumors occur at every age, from the earliest to the most advanced. Warren mentions a case which occurred in a child of ten years. M. Maisonneuve has removed them from patients of sixty-nine years of age. Flajani and Gensoul have also operated upon patients of sixty years of age. Stanley also speaks of a growth in the sinus of a child aged four years. Thus the two most opposite periods of life seem to be equally subject to this affection; hence we have reason to presume it may occur at the earliest period of infancy; for encephaloid cancers attack other parts of the body, and develop themselves with the most fearful rapidity.

The distinction we have established between the two classes has been confirmed by experience. A girl, aged fourteen, laboring under a disease of the maxillary sinus, was placed under the care of M. Guersant. The tumor, after having filled the sinus, developed itself in the nasal fossæ. The young patient was operated upon, the sinus being exposed externally, and the tumor was seized and plucked out. The microscopical examination, made by M. Broca, whose authority in this matter cannot be doubted, showed that it was formed of elements belonging to fibro-plastic tumors.

*Causes.*—The origin of these tumors cannot be ascribed to any precise cause. Dessault, Brutalour, Gensoul, &c., have observed these tumors developing themselves as a consequence of contusion of the face. A patient of Liston's affected with a tumor of this kind, had suffered from a carious tooth. If we compare these facts with those connected with the ordinary development of cancerous diseases, the agency of them would seem to be rendered doubtful.

*Mode of Development.*—We do not know whether these productions are developed most frequently in the periosteum or the mucous membrane. We are even ignorant whether the disease originates at the surface of the latter or cellular tissue of the former. Walshe inclines to the opinion that it has its origin in the mucous membrane, owing to the fact that he has seen similar productions which were developed from this tissue, designated by the name cauliflower. But whatever may be the point in the sinus from which it emanates, the morbid mass remains a long time without making any sensible progress, and is only manifested exteriorly by some functional modifications which it is presumptuous to regard as of a cancerous nature, and is most frequently accompanied by deep-seated pains of a neuralgic character. The disease often remains thus concealed for a long time in the depths of the sinus, and it is only where some mechanical injury has been inflicted that the disease becomes aggravated, increasing in malignancy, and being attended with pain of a severer and sharper kind, which the patient compares to a sensation of fullness or distension. Liston gives a case in which these symptoms were experienced. Blandin and Dupuytren give a case in which the development of the tumor was accompanied with atrophy of the surrounding tissues; the morbid growth extending to the nose. Ruysch, Acoluthus, Dessault and Canalle, have noticed such tumors protruding at the side of the mouth; Salbi, at the orbit; Flajani, from the walls of the sinus. The patient in the last case was aged sixty years; an operation was performed and followed by fatal apoplexy. The antrum was found filled with a large polypus which had destroyed the bones of the nose and orbit.

In cases of this kind fungous flesh pushes out from the jagged points, and penetrates all the cavities, forming an immense tumor which distorts the countenance and displaces the organs; thus the eye is forced from its socket, vision being destroyed or impaired, and respiration rendered difficult.

*Symptoms.*—These are local or general. Among the former we notice, impaired olfaction, involuntary flow of tears, double vision or complete loss of sight, with or without ulceration of the cornea, difficult mastication and deglutition; and finally, these tumors are insensible to the touch, but are the seat of sharp lancinating pains.

*Diagnosis of Tumors of the Maxillary Sinus.*—It is not easy to determine the seat of a disease in a region where there is so much liability to error. A tumor of the pharnyx, uvula or posterior part of the nasal fossæ, may penetrate the maxillary sinus and develop itself in this cavity towards the orbit; a case of this kind is mentioned by M. Prescot Hewett. On the other hand, a tumor of the sinus may make its way into the nose and develop itself there. Dupuytren and Blandin describe cases of this kind, which were mistaken for nasal polypi, and attempts were made to extract them. In a case described by Warren, the morbid growth forced its way into the orbit and formed a tumor under the skin. Here then is a series of circumstances, added to those already formidable, with which we have to contend in locating the seat of the disease. In order to arrive at certainty, it is necessary to be guided by much experience, and that the tumor should be explored at every accessible point. The nasal fossæ, the mouth and the pharnyx should be examined by the aid of the finger, or at least, with an instrument with which these cavities may be displayed, without wounding the parts.

By exploring these cavities, we can ascertain whether any morbid mass occupies them, and if found there, whether it is soft or enveloped in a bony covering, and yields to pressure the peculiar sensation. If this examination has been necessitated by the presence of a tumor of the superior maxillary, situated in the anterior part of the bone or even in the side of the pharnyx, and if to this there be added protrusion of the ball of the eye, no doubt will remain with regard to the seat of the tumor.

The seat of the disease having been ascertained, it still remains to determine its nature. Is the tumor liquid or solid? In ordinary cases the diagnosis is simple, but we may sometimes be deceived. Thus Gensoul mistakes dropsy of the sinus with thickening of the walls for a solid tumor. An error of the same kind was committed by M. Lawrence in an analogous case, described by Stanley. M. Ferguson had a case of dropsy of the maxillary sinus, which he confounded with encephaloid tumor. M. Bertrand cites an example of a similar mistake. To avoid error in the diagnosis, the examination should be aided with a proper exploring probe, and there are cases in which, even with this means, we may fail to arrive at absolute certainty. M. Taturn cites a tumor of the upper jaw which he had eradicated; the antrum was much dilated, and a kind of encephaloidal disease had destroyed a portion of the bone.

The distinction between soft and hard tumors being established, it remains to determine the precise nature of the disease. This is attended with more difficulty. M. A. Pettit cites the case of a child, fourteen years of age, having a tumor of the face occasioned by the kick of a horse, which was taken for a polypus. Perforation of the sinus revealed a spongy exostosis. On the other hand a tumor of the antrum may be enclosed in a bony shell, having no perforation and feeling like parchment, as observed in cysts of the jaw. It is by a thorough knowledge of all the characteristics of such morbid growths that the nature of the tumor can be ascertained. In the investigation we should avoid characteristics, common to many tumors, which are deceptive.

The consistency of the tumor, its form, its size, as well as the pain of which it is the seat, the length of time it has existed, are all circumstances which should be taken into the account in determining the nature of the disease.

The consistence of it constitutes an important characteristic; thus, fibrous and osseous tumors are hard and unyielding; their resistance is firm without elasticity. Fibro-plastic and encephaloid tumors, on the contrary, have an elasticity with marked softness in some portions of their substance.

The length of time the tumor has existed will also serve to establish the class to which it belongs; fibrous, fibro-cystic and osseous tumors are very slow in their development. Fibro-plastic and encephaloid tumors are of more rapid growth, and arriving at a certain stage of development, they double or triple their size in a short time.

After having examined the character of the tumor, and assigned it to one or other of these classes, the diagnosis may be confirmed by means of a puncturing instrument, as recommended by Professor Knes, and examining with a microscope the substance brought away.

*Prognosis.*—The prognosis of heterologous tumors is serious, particularly when they belong to the second category, or when they are of encephaloidal character; in this case, the disease always progresses, and is rarely retarded by a surgical operation, for these morbid productions have a fatal tendency to return.

As to the prognosis of fibro-plastic tumors they cannot be determined with as much certainty as in the preceding. M. M. Bennett, Bruch and Lebert, have failed to demonstrate in a satisfactory manner, in their recent enterprising researches, their benignant character. It is prudent to avoid this question.

*Comparative Prognosis.*—The comparative examination of tumors of the maxillary sinus establish some very important facts in a clinical point of view. Thus, fibrous and fibro-cartilaginous tumors are local affections which can be removed by surgical operations. Heterologous tumors, on the contrary, differ from them in their constant tendency to be reproduced after the lapse of time, of a greater or less length, after they have been removed by an operation.

It would be interesting to institute a comparison between the different kinds of tumors of the maxillary antra and at different periods of life, but the present state of pathological science does not enable us to do so.

Chapter III.—*Treatment.*—The treatment of tumors of the maxillary sinus consists in the complete eradication of their substance, either by ablation or by cauterization with caustics or the actual cautery. These operations include four methods: *extirpation*, *excision*, *cauterization*, or the *ablation*, of the organ, the seat of the disease. But these operations are not always employed, the onés are exclusive of the others. Excision and cauterization are usually resorted to in connection—the one preceding the other.

The operation by excision and cauterization is not new, but was used for the removal of tumors of the maxillary sinus as far back as the time of Acoluthers and Ruysch, (1699.) The last named writer notices, in his Observations on Surgery, the history of a case of polypus of this cavity which was cauterized with a red iron. The former cites a case of tumor, also, of this cavity, for the removal of which extirpation and cauterization were resorted to. He circumscribed the tumor with two curvilinear incisions—the one in the inside of the mouth and the other upon the external side of the jaw, and after having removed a portion of the diseased part, cauterized the remainder thoroughly with a red iron. Here both operations were employed, the one to ensure the success, excision for the removal of the tumor, and cauterization to check the hemorrhage and to destroy any portion of the morbid production that might remain.

Sometimes excision, extirpation and cauterization are all employed. But in the employment of either method, a preparatory operation is necessary to discover the seat of the disease, which consists in enlarging the opening through which the morbid production has passed, the tumor leaving no opening; it is, therefore, necessary to make a perforation into the cavity in order to reach the seat of the disease.

The fourth method, ablation or resection of the jaw, requires also cauterization to complete the operation.

These preliminaries having been gone through with, we should next consider the cases in which the interference of art is called for, and the kind of operation most proper to be performed.

Tumors of the first category, those formed of tissues analogous to normal structures, should be operated upon. But the question claiming attention before the operation, is this, whether the tumor is enclosed in the sinus as in a cyst, or whether the sinus and bone are wholly or partially implicated in the disease. In the first case, the antrum should be opened through the external wall, and if the tumor is of such a size as to require it, a portion of the bone should be cut away. In the next place, the morbid production should be removed either by excision or extirpation. The operation is then completed by touching the part from which the tumor originated with solid caustic. In the second case, if the tumor has its seat both in the antrum and jaw, as Buttner has observed, we only remove the parts implicated in the seat of the disease. If every part of the jaw is invaded by the tumor, as Hancock and Trenery have observed, then it becomes necessary to remove the entire bone.

Finally, it may be laid down as a principle, that tumors of this category should be removed by preserving as much of the bone as possible.

Are these remarks equally applicable to tumors belonging to the second category? The same distinction that we have observed in tumors of the first class should be observed here. Is it enclosed in the sinus, or does it invade a portion of the jaw? In the first case, if it is thoroughly demonstrated that the morbid growth is confined within the maxillary antrum and that it does not extend without its borders, we should follow the practice of Dessault, advised by Sabatier, and since then adopted by Blaudin; open the sinus freely, even removing a corresponding portion of the dental arch, and then destroy the diseased production by cauterizing with red iron. Weinhold proposes an entrance into the sinus by removing the dental arch; by this method, this cavity has an opening through its inferior part. This is the practice adopted by Hectuthos and Dessault in a case of polypus of the maxillary sinus which had destroyed a portion of the palatine vault.

The operation which opens the sinus through its external wall in order to remove the tumor contained there presents something very simple and seducing, but surgeons who have seen it practiced often reject it.

Lisars affirms that in the greatest number of cases that he has witnessed, the disease returns; and he adds, that in view of this, unless the entire bone is removed, the morbid production should not be interfered with. He describes, besides, the latter operation which has not since been practiced, until Gensoul, without any knowledge of Lisars' work, instituted and applied for the first time the most beautiful operation in surgery for the removal of a disease of this kind.

When the tumor has invaded the jaw as well as the antrum, the complete ablation of the bone is the only operation we would advise; this is justified by the necessity the surgeon finds in limiting the boundaries of the disease and the difficulty of exactly defining them.

I only mention as insufficient the practice advised by Weinhold and Hedenus, who wish that inflammation and suppuration of the tumor should be induced by the aid of a seton, introduced through an opening corresponding to the upper jaw, crossing the tumor and brought out through the palatine vault.

*Contra-indications.*—In the treatment of tumors of the maxillary sinus there are cases in which it is necessary for the surgeon to act, and others in which it is prudent to refrain. When the tumor is of small size and the constitution of the patient has not suffered, art should interpose, as in this case it may check the disease and prolong the life of the patient.

If the tumor is voluminous, without any constitutional impairment of the patient, if a cancerous cachexy is not developed, the surgeon's aid should still be called into requisition; first, because he may for a time check the disease which has a great tendency to progress; secondly, because he would have to do with a fibro-plastic tumor, the malignancy of which, if it be malignant, is less than that of an encephaloid tumor. Finally, when the tumor of the sinus is voluminous, extending in every direction, and the constitution of the patient is depraved, the intervention of art would only complicate a condition of things already too formidable.

